# Sea Cucumber Polysaccharides Promote Gut–Liver Axis Health by Modulating Microbiota, Metabolism, and Gene Expression in Mice

**DOI:** 10.3390/foods14172962

**Published:** 2025-08-25

**Authors:** Xue Sang, Zhuobin Xing, Boqian Zhou, Yiting Wang, Xin Guan, Fuyi Wang, Ying Li, Qiancheng Zhao, Zhibo Li

**Affiliations:** 1 College of Food Science and Engineering, Dalian Ocean University, Dalian 116023, China; sangxue116@163.com (X.S.);; 2 School of Food Science and Technology, Dalian Polytechnic University, Dalian 116034, China; 3 Dalian Key Laboratory of Marine Bioactive Substances Development and High-Value Utilization, Dalian 116023, China; 4 Liaoning Provincial Marine Healthy Food Engineering Research Centre, Dalian 116000, China; 5 Collaborative Innovation Center of Provincial and Ministerial Co-Construction for Marine Food Deep Processing, Dalian Polytechnic University, Dalian 116034, China

**Keywords:** sea cucumber polysaccharides, metabolomic analysis, prebiotic effects, arginine biosynthesis

## Abstract

This study investigated the beneficial effect of sea cucumber polysaccharides (SCP) on gut microbiota composition, metabolic profiles, and liver gene expression in mice. Using an integrative approach combining microbiome, metabolome, and transcriptome analyses, we demonstrated that SCP supplementation led to a marked rise in *norank_f_Muribaculaceae* levels and reduced the Firmicutes-to-Bacteroidota ratio. Metabolomic analysis revealed key alterations in amino acid and lipid metabolism, with L-arginine and 7-dehydrocholesterol identified as potential mediators of SCP’s beneficial effects. Transcriptomics revealed genes expression across nine metabolic pathways, with genes involved in steroid biosynthesis being upregulated, while those related to protein digestion and absorption were downregulated. Spearman’s correlation analysis highlighted strong associations between gut microbiota, lipid metabolism-related genes, and corresponding metabolites. Integration omics data further suggested that SCP primarily supports arginine biosynthesis through gut–liver axis crosstalk. These results provide an important basis for developing SCP-based functional food with prebiotic properties to support metabolic and liver health.

## 1. Introduction

The demand for sea cucumbers, valued for their edibility and medicinal properties, has led to rapid growth in China’s sea cucumber industry. In 2024, the industry reported a total production of 292,045 tonnes [1]. As a high-value seafood, sea cucumber has attracted attention for its health benefits, particularly in the wake of recent epidemics. It is gradually transitioning from a traditional ingredient to a functional health food, containing a variety of functional food ingredients, with polysaccharides second only to protein in abundance, and they are among the most important active substances in sea cucumbers [2]. Most polysaccharides cannot be directly digested and absorbed by the human body. However, they can safely pass through the digestive tract and reach the distal intestine, where they interact with the gut microbiota [3,4,5]. As a carbon source for intestinal bacteria, polysaccharides promote the growth of beneficial microbes and exert prebiotic effects by modulating the gut microbiota and altering microbial diversity through their metabolic byproducts. In recent years, studies have shown that using prebiotics to regulate the gut microbiota can alleviate mild intestinal inflammation and enhance gut barrier integrity, thereby improving metabolic balance. Polysaccharides play a key role in modifying and reshaping the diversity and composition of the gut microbiota. Additionally, they are degraded by microbial fermentation, generating bioactive metabolites that further influence host health. Emerging research demonstrates that sea cucumber-derived sulfated polysaccharides can modulate energy metabolism and counteract metabolic dysfunctions resulting from excessive nutrient intake [6]. Current studies on the activity of sulfated polysaccharides from sea cucumber have primarily focused on the regulation of glucolipid metabolism.

As research progresses, scholars have discovered significant variations in the chemical composition of polysaccharides derived from different sea cucumber species. These differences, which include monosaccharide composition, glycosidic bond types, molecular weight, and the presence of critical sulfate groups, directly influence their bioactive properties [2]. Utilizing sulfated polysaccharides from the waste liquid produced during commercial processing of sea cucumbers offers high economic value, as it prevents environmental pollution and avoids wasting biological resources. Sulfated polysaccharides marine have also been shown to regulate gut microbiota, affecting the host’s health status through their metabolites [7]. Our earlier studies showed that sulfated polysaccharides obtained by boiling the sea cucumber *Apostichopus japonicus* in water can inhibit obesity through gut microbiota modulation and metabolic profile alterations in mice fed a high-fat diet [6], although further investigation into their roles in healthy bodies and the underlying mechanisms is still needed.

While global dietary guidelines are largely consistent, the focus on gut microbiota from a dietary perspective has only just beginning. Combining a deep understanding of the role of gut microbiota in host health with targeted foods represents a potential opportunity to improve dietary health for all. Understanding the interplay between gut microbiota and host health can lead to innovative dietary approaches that prioritize microbial needs while conferring beneficial effects to the host. Intestinal microbe-directed ingredients hold promise for future healthy food development, as evidenced by studies showing increased abundance of specific bacterial strains in mice treated with sea cucumber-derived sulfated polysaccharides [2]. One of the main ways in which the intestinal flora interacts with its host is through small molecules produced as intermediate or final products of microbial metabolism. The effect of SCP on metabolites of intestinal microorganisms, such as lipopolysaccharide, short-chain fatty acids, and bile acids, has emerged as a prominent research priority. The mechanism by which intestinal flora is regulated varies according to the different structures of sea cucumber sulfated polysaccharides [7]. Thus, understanding how intestinal flora metabolizes specific polysaccharides, microbial community interactions, and their metabolite profiles is crucial. Moreover, a number of other studies have suggested that the metabolic improvements induced by dietary intake of sulfated polysaccharides intake are not only triggered by gut microbiota and their metabolites but may result from the synergistic action of multiple pathways [8]. Recent developments in transcriptome technology have drawn attention to the role of mRNA in the mechanisms behind changes in intestinal flora. It has been shown that dietary interventions in animal models can affect some mRNA expression levels [9]. Based on the above findings, the association between changes in intestinal flora and metabolites induced by SCP intervention, along with alterations in mRNA expression, warrant further investigation.

Single-omics techniques, such as microbiome, metabolome, and transcriptome analyses, are well-established tools applied across diverse research domains. Integrating multi-omics approaches enables the discovery of novel biomarkers for disease prevention and diagnosis, offering a comprehensive systemic view. This study employed a combined multi-omics strategy to investigate how SCP influences murine metabolic pathways, gene expression dynamics, and intestinal microbial balance.

## 2. Materials and Methods

### 2.1. Preparation of SCP

Sea cucumbers (*Apostichopus japnoicus*), originally captured from the Dalian Bohai Sea, were supplied by Provided by Xinyulong Marine Biological Seed Industry Technology Co., Ltd., Dalian, China. The body walls of the sea cucumbers were boiled in hot water (85–90 °C) for 15 min and the resulting liquid, which contained polysaccharides was extracted with three volumes (*v*/*v*) of anhydrous ethanol and stored at 4 °C for 24 h. The mixture was then centrifuged at 8000× *g* for 15 min and the pellet was retained and lyophilized for subsequent extraction of polysaccharides according to a previously described methodology [10]. The final preparation of polysaccharides was designated as SCP (sea cucumber polysaccharides).

The structural characterization of SCP, including quantification of sulfated groups, uronic acid, and protein contents, along with the determination of monosaccharide profile and the relative molecular weight distribution, was performed according to the analytical methodologies established in our previous investigation [6].

### 2.2. Animals and Experimental Design

Eighteen male C57BL/6J mice (specific pathogen-free, aged six weeks) were obtained from Changsheng Biotechnology Co., Ltd., Benxi, China (License No. SCXK2020-0001). The animals were housed in ventilated cages (three per cage) under controlled conditions: 12-h light/dark cycle, temperature maintained at 22 ± 2 °C, and relative humidity of 50 ± 10%. All efforts were made to minimize animal suffering and reduce the number of animals used. After being acclimatized for seven days, the animals were randomly divided into two groups: a control group and an SCP group, with nine mice per group. Mice in the SCP group received daily oral administrations of SCP (400 mg/kg/day), whereas control group animals were given an equal volume of distilled water [11,12]. The treatment lasted for 8 weeks, and during this period, the mice were fed a normal diet. Food intake and body weight were measured daily. After 8 weeks, all mice were fasted for 12 h before being anesthetized with ethyl ether. Blood was collected from each anesthetized animal via the orbital vein. The mice were then sacrificed, and their liver, epididymal fat, cecum, and colon were precisely removed and weighed. All animal experiments in this study complied with the ethical standards outlined in the National Research Council Guidelines and received formal approval from Obio Technology Corp., Ltd.’s Institutional Animal Care and Use Committee, Shanghai, China (IACUC Approval No. 112).

### 2.3. Biochemical Analysis of Lipid-Related Compounds in Serum

Total cholesterol (TC), triacylglycerols (TG), high-density lipoprotein cholesterol (HDL-C), low-density lipoprotein cholesterol (LDL-C), and malondialdehyde (MDA) were measured using commercial kits (Jiancheng Bioengineering Institute, Nanjing, China). These experiments were performed according to the manufacturer’s instructions.

### 2.4. Histological Examination

Freshly isolated liver and epididymal adipose tissue were fixed with paraformaldehyde. They were then dehydrated, embedded in paraffin, and cut into 5-μm thick slices. The slices were stained with hematoxylin and eosin and imaged under a light microscope (Leica Instruments Ltd., Wetzlar, Germany).

### 2.5. Gut Microbiota Analysis

The analysis of gut microbiota was performed by Shanghai Majorbio Bio-pharm Technology Co., Ltd. (Shanghai, China). Briefly, bacterial genomic DNA was isolated from cecal samples using an E.Z.N.A.^®^ Soil DNA Kit Manufactured by Omega Bio-tek, Inc., Norcross, GA, USA. and quantitative analysis was performed using a NanoDrop spectrophotometer from Manufactured by Thermo Fisher Scientific, Wilmington, NC, USA. The 16S rRNA gene V3–V4 region was amplified using barcoded primers 338F/806R. Libraries were prepared with the NEXTFLEX Rapid DNA-Seq Kit Manufactured by Bioo Scientific Corporation, Austin, TX, USA. and sequenced on an Illumina MiSeq PE300 platform. OTUs were clustered at 97% similarity (Uparse v7.0), and taxonomy was assigned via RDP Classifier (v2.2, confidence threshold: 0.7) against the 16S rRNA database.

### 2.6. Gut Metabolites Analysis

As described in our previous study [6], the methods can be briefly summarized as follows: cecal metabolites were extracted from 50 mg samples using 400 µL methanol/water (4:1, *v*/*v*) with homogenization (50 Hz, 6 min), vortexing, sonication (40 kHz, 30 min, 5 °C), and protein precipitation (−20 °C). After centrifugation (13,000× *g*, 15 min, 4 °C), the supernatant was analyzed via a Thermo UHPLC-Q Exactive HF-X system with an ACQUITY UPLC HSS T3 column (100 × 2.1 mm, 1.8 µm; 40°C) at 0.4 mL/min. Mobile phases: (A) 0.1% formic acid in water/acetonitrile (95:5); (B) 0.1% formic acid in acetonitrile/isopropanol/water (47.5:47.5:5). The gradient elution was 0–24.5% B (0–3.5 min), 24.5–65% B (3.5–5 min), 65–100% B (5–5.5 min), held (5.5–7.4 min), then re-equilibrated (7.4–10 min). ESI parameters: ±3500 V ion-spray voltage, 425 °C heater, 325 °C capillary, 60,000 (MS)/7500 (MS/MS) resolution, and *m*/*z* 70–1050 scan range. Data were acquired in DDA mode (20–60 eV collision energy) and processed using Progenesis QI 2.3 for peak alignment (RT, *m*/*z*, intensity). Metabolites were identified via HMDB and Metlin databases.

Principal component analysis (PCA) was performed using the ropls package (v 1.6.2) in R software 4.5.1. Variables were considered statistically significant if the variables importance in projection (VIP) > 1 and *p* < 0.05. Differential metabolites were defined as those with *p* < 0.05 and an absolute log2 fold change (FC) greater than 1.0 or 1.2. Based on the KEGG database (Kyoto Encyclopedia of Genes and Genomes, http://www.genome.jp/kegg, accessed on 3 February 2024), relevant metabolic and signal transduction pathways were analyzed for enrichment.

### 2.7. Transcriptome Sequencing of Liver Tissue

The liver samples in the control and SCP groups were selected for transcriptome analysis, 3 parallel per group. Total RNA was extracted from the tissue using TRIzol^®^ Reagent (Invitrogen, Carlsbad, CA, USA), and genomic DNA was removed using DNase I (TaKara, Beijing, China). RNA quality was determined with a 2100 Bioanalyser (Agilent, Santa Clara, CA, USA) and quantified with the ND-2000 (NanoDrop Technologies, Shanghai, China) to ensure that qualified samples were used for transcriptomic analysis (OD_260/280nm_ = 1.8–2.2, OD_260/230nm_ ≥ 2.0, RIN ≥ 6.5, 28S:18S ≥ 1.0, >1 μg). RNA-seq transcriptome libraries were prepared with the TruSeqTM RNA sample preparation Kit from Illumina San Diego, CA, USA, using 1 μg of total RNA. Messenger RNA was isolated by the polyA selection method with oligo(dT) beads and fragmented. Double-stranded cDNA was synthesized using a SuperScript double-stranded cDNA synthesis kit (Invitrogen, Carlsbad, CA, USA) with random hexamer primers (Illumina, San Diego, CA, USA). End repair was conducted, followed by the addition of an A base to the 3’ end and ligation of Y-shaped adapters. Subsequently, PCR amplification was performed, and DNA purification magnetic beads were used to isolate fragments ranging from 200–300 bp. After quantification with TBS380, the cDNA libraries were sequenced was sequenced with the Illumina HiSeq xten/NovaSeq 6000 sequencer (2 × 150 bp read length).

### 2.8. Data Analysis of RNA-Seq and Bioinformatics Analyses

After quality control, clean reads were aligned against the reference genome using HISAT2 (http://ccb.jhu.edu/software/hisat2/index.shtml, accessed on 7 February 2024) to obtain mapped data for subsequent transcript assembly, expression calculation, etc., and the results were compared for quality assessment, mainly including sequencing saturation, gene coverage, and reads distribution. Transcript abundance was calculated from the number of reads mapped to a genomic region (reads counts) as revealed by RNA-Seq analysis. After obtaining the number of reads, the differences in gene expression between the control and SCP group samples were analyzed to identify the genes that were differentially expressed as a result of SCP treatment. Transcripts meeting the thresholds of Q ≤ 0.05 and |log_2_FC| > 1 were defined as statistically significant. Subsequent KEGG enrichment analysis highlighted metabolic pathways with Bonferroni-significant DEGs (*p* ≤ 0.05), executed using KOBAS (http://kobas.cbi.pku.edu.cn/home.do, accessed on 7 February 2024) [13].

### 2.9. Statistical Analysis

Data are expressed as mean ± SD and analyzed using GraphPad Prism 5.01 (GraphPad Software, 10.5.0, USA). One-way ANOVA with Tukey’s post hoc test was applied, with statistical significance set at *p* < 0.05. Spearman correlations were visualized using R (v3.3.1) heatmap package.

## 3. Results

### 3.1. Quantification of Bioactive Compounds in SCP

*Apostichopus japonicus* polysaccharides (SCP) extracted by boiling the sea cucumbers in water were found to have the following compositional characteristics: 22.82 ± 0.01% sulfate group content, 13.42 ± 0.10% uronic acid content, and 12.53 ± 0.01% protein content. Monosaccharide composition analysis revealed the presence of mannose, glucosamine, glucuronic acid, Glucose, N-acetyl-D-galactosamine, galactose, and fucose in a molar ratio of 4.89:7.74:1.09:4.61:3.53:8.89:69.26. SCP exhibited a relative molecular mass of 519.11 kDa.

### 3.2. Impact of SCP on Body/Organ Mass, and Lipid Metabolism in Mice

**Figure 1:**
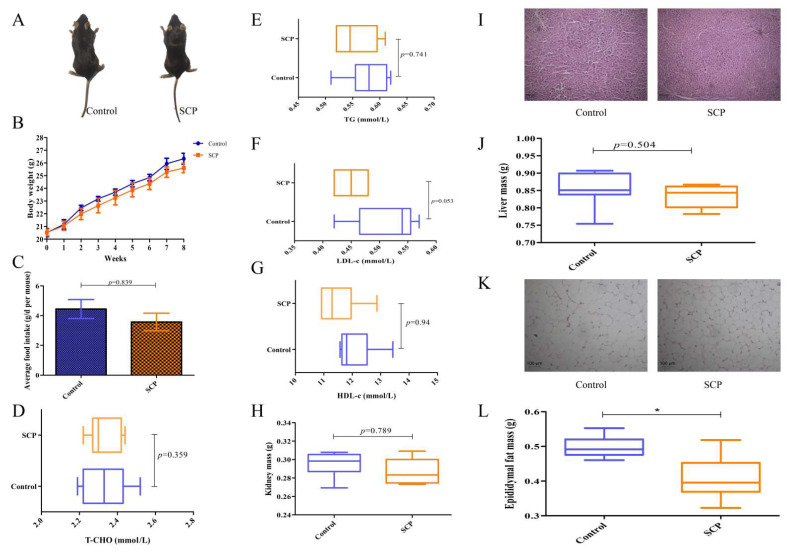
Dietary SCP affects body weight gain, fat accumulation, and lipid metabolism-related indices in normal mice. (**A**) Mice morphology; (**B**) body weight; (**C**) average food intake; (**D**–**G**) serum T-CHO, TG, LDL-c, and HDL-c contents; (**H**) kidney mass; (**I**) liver morphology, representative H&E staining images of the liver (200×); (**J**) liver mass; (**K**) epididymal fat morphology, representative H&E staining images of the epididymal fat (200×); and (**L**) epididymal fat mass. Data are expressed as mean ± SD (n = 9). Graph bars marked by asterisks represent statistically significant differences (*p* < 0.05) based on one-way analysis of variance (ANOVA) with Tukey’s multiple comparisons test. * *p* < 0.05.

To assess the effect of SCP on the phenotype and hepatic steatosis, mice were fed normal diets with or without SCP supplementation for eight weeks. At the conclusion of the experiment, control group mice exhibited soft and fine dorsal fur, while those in the SCP group demonstrated improved coat quality, characterized by enhanced texture and sheen (Figure 1A). After eight weeks, SCP-treated mice demonstrated reduced body weight and lower total food intake compared to controls, though these differences lacked statistical significance. This observation suggests that SCP’s potential weight-modulating effects may be mediated through decreased food consumption (Figure 1B,C).

As shown in Figure 1D–G, SCP administration showed no significant effects on serum lipid profiles or organ weights (liver/kidney) compared with controls. However, it significantly decreased weight of epididymal fat (*p* < 0.05), aligning with its observed impact on body weight (Figure 1H,J,L). H&E staining of the liver and epididymal fat was conducted to assess the impact of SCP on their histological and morphological features. The results indicated that SCP intervention had little effect on hepatic vesicles but decreased lipid droplets (Figure 1I,K). Thus, SCP exerted some beneficial effects on normal mice and showed potential for fat loss.

### 3.3. Impact of SCP on Gut Microbiome

**Figure 2:**
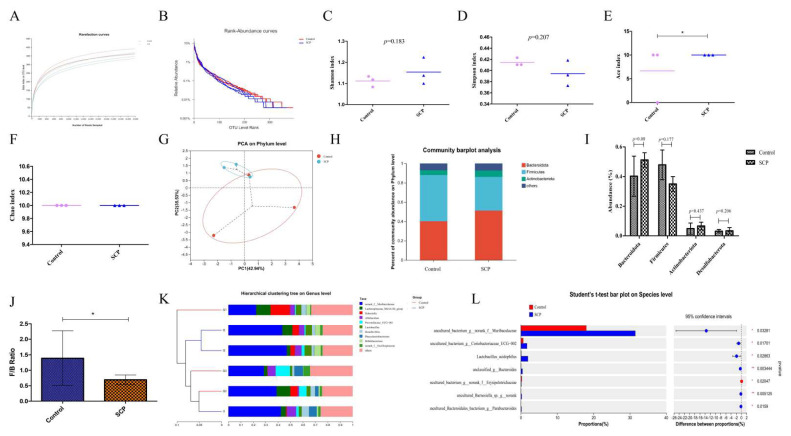
The effects of SCP intervention on gut microbiota in mice. (**A**) Rarefaction curves; (**B**) rank-abundance curves; (**C**) Shannon index; (**D**) Simpson index; (**E**) ACE index; (**F**) Chao index; (**G**) PCoA on phylum level; (**H**) relative abundance at phylum level; (**I**) abundance of major phylum; (**J**) the ratio of Firmicutes to Bacteroidota; (**K**) hierarchical clustering tree on genus level; and (**L**) Student’s *t*-test bar plot on species level. Data are expressed as mean ± SD (n = 3). Graph bars marked with * on top represent statistically significant differences (*p* < 0.05) based on one-way analysis of variance (ANOVA) with Tukey’s multiple comparisons test. * *p* < 0.05.

In our experiment, each treatment group of nine mice was caged in three cages. To make the cecum microbiome more representative, we mixed the cecal contents of mice from each cage evenly and measured them as one sample. The OTU rarefaction curves for both the control and SCP groups plateaued with increasing sampling depth, suggesting adequate and well-balanced sequencing coverage (Figure 2A). This study employed a sufficient number of sequenced samples to characterize the microbial community. Rank-abundance curves illustrated the species abundance and evenness in both control and SCP groups (Figure 2B). SCP had no statistically significant effect on Shannon, Simpson or Chao indices, except for ACE index (*p* < 0.05). Alpha diversity reflects the richness and diversity of species in a specific ecosystem, Shannon and Simpson indices reflect species richness, while ACE and Chao indices reflect species abundance.

Principal component analysis (PCA) demonstrated a separation between the control and SCP groups, suggesting that SCP might exert a regulatory effect on the gut microbiota, but this effect was not statistically significant (Figure 2G).

Following SCP intake, certain changes in the gut microbiota composition of mice became evident (Figure 2H). At the phylum level, Bacteroidota and Firmicutes dominated the microbial community, followed by Actinobacteriota and Desulfobacterota (Figure 2H,I). These findings indicate a similar gut microbiota profile between the two mouse groups, aligning with our prior research [6]. However, the SCP group had a significantly lower Firmicutes/Bacteroidota (F/B) ratio than the control group (Figure 2J).

The gut microbiota composition in both control and SCP-treated mice was dominated by several genera, including *norank_f__Muribaculaceae*, *Lachnospiraceae_NK4A136_group*, *Dubosiella*, *Allobaculum*, *Prevotellaceae_UCG-001*, *Lactobacillus*, *Desulfovibrio*, *Phascolarctobaterium*, *Bifidobacterium* and *norank_f_Oscillospiraceae*, as illustrated in Figure 2K. Among these, *norank_f__Muribaculaceae* levels increased significantly (*p* < 0.05) with an increase in the dose of SCP administered. Intergroup Student's *t*-test analysis revealed significa nt differences in species composition between control and SCP groups (Figure 2L). Notably, SCP dietary supplementation substantially enhanced the abundance of *uncultured_bacterium_g_norank_f_Muribaculaceae*, *uncultured_bacterium_g_Coriobacteriaceae_UCG-002*, *Lactobacillus_acidophilus*, *unclassified_g_Bacteroides*, *uncultured_Barnesiella_sp._g_norank*, and *uncultured_Bacteroidales_bacterium_g_Parabacteroides*, while significantly decreasing the levels of *uncultured_bacterium_g_norank_f_Erysipelotrichaceae*.

### 3.4. Impact of SCP on Metabolic Pathway Modifications

**Figure 3:**
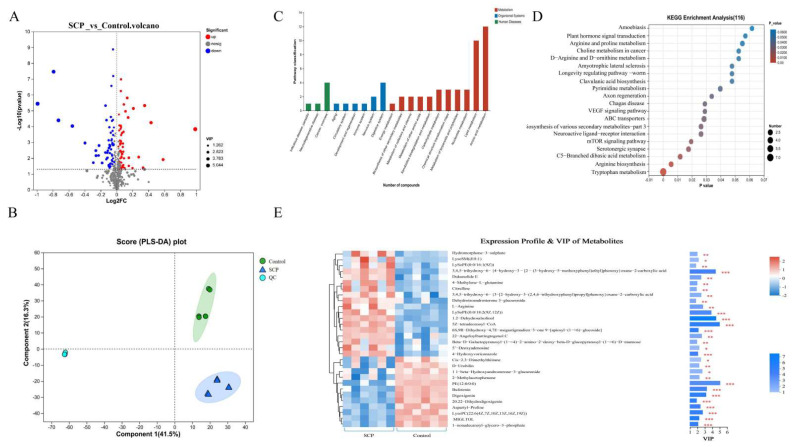
Effect of SCP intervention on cecal content metabolites between SCP and Control groups. (**A**) Volcano plots of altered metabolites with *p* < 0.05 and |log2FC| > 1.2 between Control and SCP groups (n = 6); (**B**) partial least squares discriminant (PLS-DA) analysis; (**C**) differences in KEGG functional pathways; (**D**) KEGG enrichment pathway analysis; (**E**) VIP bar graph. The top 30 differential metabolites were identified between SCP and Control groups. On the far right of the picture is the *p* value. * *p* < 0.05, ** *p* < 0.01 and *** *p* < 0.001 indicated significant correlation.

We performed non-targeted metabolomic profiling of cecal contents using LC-MS, analyzing 6 samples each from the SCP and control group (total n = 12). The study identified 1031 metabolites in positive ion mode and 1244 in negative ion mode through primary and secondary mass spectrometry data analysis. Compared to controls, the SCP group showed significantly more up-regulated than down-regulated metabolites (Figure 3A). The partial least squares discriminant analysis (PLS-DA) score chart is commonly used to intuitively show the classification effectiveness of the model. As shown in Figure 3B, intestinal metabolites were significantly different between the SCP and control groups. By comparing the two groups, the intervention of SCP was found to lead to differences in metabolites, and the mice in the two groups were clearly separated into different clusters.

KEGG functional analysis of differential metabolic pathways revealed that within organismal systems, these pathways were primarily linked to the digestive system, with a predominant focus on amino acid and lipid metabolism, and these pathways were predominantly associated with cancer (Figure 3C). Relative to the KEGG pathway database (Figure 3D), tryptophan metabolism and arginine biosynthesis were significantly enriched (*p* < 0.01). Analysis of metabolite differences between the SCP and control groups, along with their contributions to group separation (Figure 3E), identified five key metabolites with the greatest impact. These were PE (12:0/0:0), 5Z-tetradecenoyl-CoA, 1,2-dehydrosalsolinol, 3,4,5-trihydroxy-6-{4-hydroxy-3-[2-(3-hydroxy-5-methoxyphenyl)ethyl]phenoxy} oxane-2-carboxylic acid (3,4,5-trihydroxy-6-XX) and LysoPE (0:0/18:2(9Z,12Z)). Our findings revealed distinct metabolic pathway variations when comparing the SCP-administered group with controls.

### 3.5. Impact of SCP on the Hepatic Transcriptome

**Figure 4:**
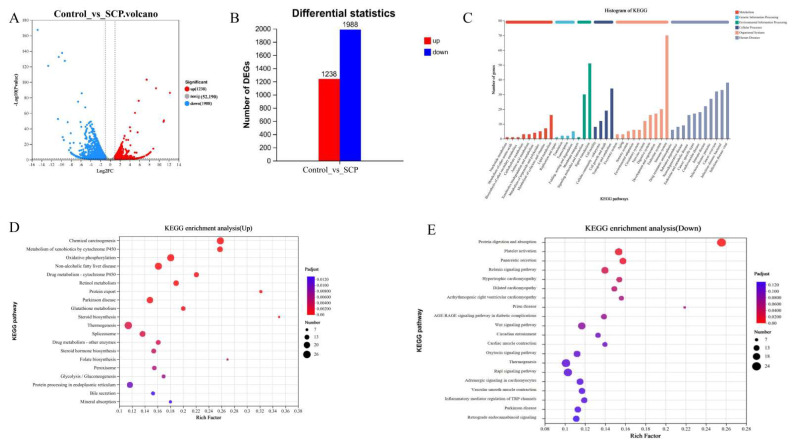
Transcriptome analysis of SCP and control groups (n = 3). (**A**) Volcanic map of DEGs; (**B**) statistics of up-regulated and down-regulated genes; (**C**) functional annotation of DEGs; (**D**) KEGG pathway enrichment analysis of up-regulated genes; (**E**) KEGG pathway enrichment analysis of down-regulated genes.

To assess the probiotic effects of SCP supplementation in healthy mice, liver tissue transcriptomes from both the control and SCP-treated groups were analyzed. Analysis revealed 3226 differentially expressed genes (DEGs), with 1238 showing increased expression and 1988 exhibiting decreased expression (Figure 4A,B). KEGG pathway enrichment of DEGs revealed nine metabolism-associated pathways (including lipid metabolism and amino acid metabolism), four pathways associated with genetic information processing, three with environmental information processing, four with cellular processes, ten with organismal systems, and eleven linked to human diseases based on functional annotation (Figure 4C). Additionally, KEGG enrichment analysis demonstrated that up-regulated genes were predominantly enriched in steroid biosynthesis, protein export, chemical carcinogenesis, and metabolism of xenobiotics by cytochrome P450 (*p* < 0.001, Figure 4D). The down-regulated genes showed predominant enrichment in protein digestion and absorption pathways (*p* < 0.001, Figure 4E).

### 3.6. Interrelationships Among Microbiome, Metabolome, and Transcriptome

#### 3.6.1. Associations Between Body/Organ Weight Parameters, Lipid Metabolism-Related Indices, and Microbial Composition

**Figure 5:**
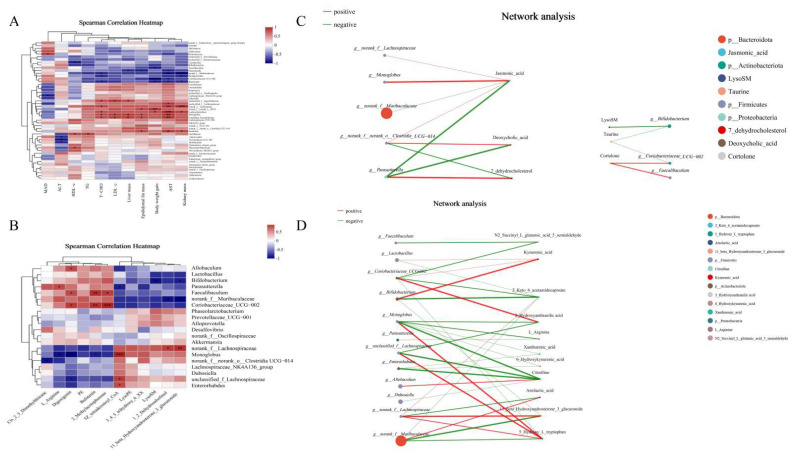
Correlation analyses of the gut microbiome and metabolites with lipid metabolism-related indices associated with SCP supplementation. (**A**) Spearman’s correlation analysis of gut microbiome and lipid metabolism-related indices, (**B**) Spearman’s correlation analysis of obesity-related gut microbiome and metabolites, (**C**) network analysis of gut microbiome and metabolites in lipid metabolism pathway, (**D**) network analysis of gut microbiome and metabolites in amino acid metabolism pathway. The size of the node indicates the size of genus or metabolites abundance, and different colors indicate different genus or metabolites, the color of the connecting line indicates positive and negative correlation, red indicates positive correlation and green indicates negative correlation; the thickness of the line indicates the size of the correlation coefficient, and the thicker the line is, the higher the correlation between the species is, and the more lines there are, the closer the connection between the nodes is. * *p* < 0.05, ** *p* < 0.01, *** *p* < 0.001.

We also performed correlation analysis between the altered microbiome and various physiological and biochemical indices. The measured indices comprised body weight gain, organ masses, and serum parameters. *Parasutterella* demonstrated significant negative correlations with epididymal fat mass, liver mass, and AST levels, suggesting its potential role in reducing fat accumulation and possibly protecting against liver injury. By contrast, *norank_f_norank_o_RF39*, *Monoglobus*, *Candidatus_Saccharimonas*, and *Roseburia* were positively correlated epididymal fat mass, body weight gain, liver mass, and AST, indicating that they could be instrumental in the process of fat accumulation (Figure 5A). These results demonstrate that SCP could be utilized by gut microbes, thereby modifying the organ weight and playing a beneficial role in alleviating fat accumulation and liver damage.

#### 3.6.2. Correlation Analysis Between Microbiome and Metabolome

To examine the relationship between gut microbiota and metabolites, we analyzed correlations between the top 20 genera (selected via community heatmap analysis) and metabolites that met our screening criteria (*p* < 0.05 with |log2FC| > 1.2), using data from both SCP and control groups (Figure 5B). L-arginine (organic acids and derivatives) showed a significant positive correlation with *Parasutterella* abundance but a negative correlation with *Monoglobus* abundance. The primary gut bacterial genus *Bifidobacterium* exhibited a strong inverse association with LysoSM and 11-beta-hydroxyandrosterone-3-glucuronide (organoheterocyclic compounds). The relative abundance of potential probiotic *Alloprevotella* showed a significant positive correlation with digoxigenin (lipids and lipid-like molecules). *Faecalibaculum* and *Coriobacteriaceae_UCG-002* were positively correlated with bufotenine (*organoheterocyclic* compounds), digoxigenin and 2-methylacetophenone (organic oxygen compounds). These findings may offer novel insights into how dominant gut microbiota confer benefits via metabolites.

As the enriched differential metabolites consisted mainly of those derived from amino acids and lipid metabolisms, further mechanistic study was performed to clarify the underlying probiotic effect of SCP. The correlation network linking intestinal bacteria with amino acid and lipid metabolism-related metabolites was analyzed to achieve this (Figure 5C,D). The results revealed statistically significant links (*p* < 0.05) between certain intestinal bacterial groups and diverse metabolic compounds. Notably, *norank_f_Muribaculaceae*, *Monoglobus*, *norank_f_Lachnospiraceae*, *Parasutterella*, *Bifidobacterium*, *Coriobacteriaceae_UCG-002*, and *Faecalibaculum* occupied key nodes in the network, demonstrating strong associations with multiple metabolites involved in amino acid and lipid metabolism, e.g., 2-keto-6-acetamidocaproate, L-arginine, citrulline, 11-beta-hydroxyandrosterone-3-glucuronide, jasmonic acid and 7-dehydrocholesterol. Thus, SCP exerted its beneficial effects through gut microbiota-driven metabolites.

#### 3.6.3. Analysis of Correlations Between Gut Microbiota and Liver Transcriptomes

**Figure 6:**
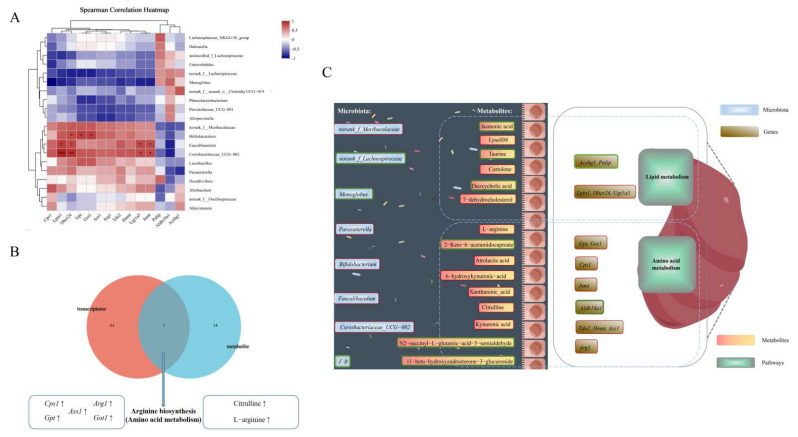
Correlation analysis amongst microbiome, metabolome and transcriptome. (**A**) Spearman’s correlation of genus-level gut microbiota with differential genes. (**B**) Combined metabolome and transcriptome analysis. (**C**) Schematic o5f the mechanism by which SCP exerts its beneficial effects, red boxes represent upregulation, and green boxes represent downregulation. * *p* < 0.05, ** *p* < 0.01, *** *p* < 0.001.

We performed a correlation analysis between gut microbiota and liver transcriptome to assess their relationship. Spearman’s correlation assessed associations between gut microbial genera and key genes for amino acid/lipid metabolism. As shown in Figure 6A, *Faecalibaculum* and *Coriobacteriaceae_UCG-002* were significantly and positively correlated with *Lpin1*, *Dhcr24*, *Ugt1a5* and *Inmt*. *Bifidobacterium* was significantly and positively correlated with *Dhcr24*, *Gpt* and *Got1*. Thus, these gut microbes and genes may play a key role in amino acid and lipid metabolic pathways.

#### 3.6.4. Combined Metabolome and Transcriptome Analysis

A combined analysis of the transcriptome and metabolome was conducted to establish the relationship between DEGs and metabolites. As depicted in Figure 6B, 61 KEGG pathways at the transcriptional level and 14 KEGG pathways at the metabolome level exhibited changes. Among them, arginine biosynthesis, a pathway associated with amino acid metabolism, was found to be common to both the transcriptome and metabolome datasets. The related genes *Cps1*, *Gpt*, *Ass1*, *Arg1* and *Got1*, along with the metabolites citrulline and L-arginine, were up-regulated. These results demonstrated that SCP exerted its beneficial effects mainly by regulating arginine biosynthesis. 

## 4. Discussion

The functional food market has been developing rapidly in response to an increased level of health awareness among the public. Among the different kinds of health foods, polysaccharide functional foods are attracting attention because of their unique health functions and wide range of applications. Through assessment of multiple physiological indicators in response to SCP administration in mice, this study identified SCP’s positive influence on core phenotypic characteristics. These findings enable subsequent investigation of SCP’s probiotic mechanisms through integrated microbiome, metabolome, and transcriptome analyses.

Mice given SCP intake did not show significant changes in body weight or lipid metabolism-related indices. Interestingly, these mice displayed significantly lower epididymal fat weight and reduced formation of lipid droplets, suggesting an anti-obesity potential linked to SCP intake. This hypothesis received support from intestinal microbial community findings. The SCP group exhibited minimal alterations in gut microbiota composition versus controls. In addition, its microbiota consisted of higher Bacteroidota abundance whereas the microbiota of the control group was richer in Firmicutes abundance. The SCP group exhibited a markedly lower Firmicutes/Bacteroidota (F/B) ratio than the control, implying that SCP-mediated gut microbiota modulation could help mitigate diet-induced obesity and sustain a lean phenotype in mice. This finding was confirmed in our previous study [6].

Recently, gut microbiota has been recognized as a key environmental factor in the pathogenesis of various diseases. Certain polysaccharides can promote the growth of beneficial gut bacteria while suppressing pathogenic microbes, thereby helping prevent disease [14]. *Muribaculaceae* is a functionally diverse and important group of intestinal flora. These bacteria play a key role in regulating the energy balance, maintaining intestinal function, and preventing disease through energy production and transfer, the abundance of *Muribaculaceae* in mouse intestine has been shown to increase significantly after the intervention of cordyceps polysaccharides [15]. *Coriobacteriaceae_UCG-002* possesses a strong ability to hydrolyze bile and can synthesize a variety of hydroxylated steroid dehydrogenases involved in bile acid metabolism, lipid processing, and weight regulation [16]. *Lactobacillus* species differentially influence host-specific body weight alterations, *Lactobacillus acidophilus* has been shown to alleviate obesity in mice by regulating gut microbiota dysbiosis and improving intestinal permeability [17]. *Bacteroides*, fa prevalent genus in the human gut, engage in a symbiotic relationship with their host, aiding in food breakdown and the production of essential nutrients and energy. For example, *Bacteroides thetaiotaomicron* levels are significantly lower in obese individuals and are associated with fat metabolism [18]. *Barnesiella* is a key protective gut bacterium that removes harmful bacteria and can regulate intestinal homeostasis through TLR-4 signaling [19]. *Erysipelotrichaceae* is correlated with inflammation, and its abundance has been shown to be downregulated after an inulin diet in mice [20]. *Bacteroidales* are considered to be key members of the human colonic microbiota. They can break down diverse dietary polysaccharides and, by generating metabolites like short-chain fatty acids, help maintain intestinal mucosa integrity and immune defenses [21].

The present study produced consistent results. To strengthen these findings, we performed Spearman’s correlation analysis on microbial genera (Figure 5A). The genera *norank_f_Muribaculaceae*, *Coriobacteriaceae_UCG-002*, *Lactobacillus*, *Parabacteroides*, and *Bacteroides* exhibited significant negative correlations with body weight, organ weight, and lipid metabolism-related indices. Therefore, SCP may support health by regulating the abundance of these gut microbes, manifesting its probiotic effects on mice.

2-keto-6-acetamidocaproate is an intermediate lysine catabolism that is further metabolized to form acetyl coenzyme A, which enters the tricarboxylic acid cycle and provides cellular energy [22]. Citrulline, an intermediate in the urea cycle, helps scavenge ammonia and converts it to L-arginine, enhancing nitric oxide production, cardiovascular function, and immune response [23]. It has also been shown that microbially produced L-citrulline and its conversion to L-arginine are key regulator of skeletal adaptations, improving outcomes in aged and de-ovulated mice [24].

Abnormal levels of androgen metabolites, including 11-beta-hydroxyandrosterone-3-glucuronide, may reflect disturbances in androgen metabolism affecting fat distribution and metabolism. Low levels of androgens or their metabolites have been found to associate with visceral fat accumulation, insulin resistance and metabolic syndrome [25]. Jasmonic acid, a plant oxylipin, is structurally similar to prostaglandins, and prostaglandin E2 exacerbates obesity-related disorders by promoting adipose tissue inflammation and insulin resistance [26]. 7-dehydrocholesterol, an intermediate in the distal cholesterol biosynthesis pathway, inhibits ferroptosis, utilizes conjugated dienes, exerts antiphospholipid autoxidation, and protects cytoplasmic and mitochondrial membranes [27].

The transcriptomic data showed that nine pathways, including lipid metabolism and amino acid metabolism, were associated with metabolism (Figure 4C), consistent with the metabolomics results. Biomarkers of lipid metabolism include *Lpin1*, *Dhcr24*, *Acsbg1*, *Ugt1a5*, and *Pnlip*. *Lpin1* can act as a transcriptional cofactor that regulates fatty acid oxidation and lipogenesis and is upregulated in certain types of cancer cells, and its activation of phosphatidic acid phosphatase is required for the survival of these cancer cells [28]. The *Dhcr24* gene is one of the major target genes that is associated with Alzheimer’s disease and type 2 diabetes through regulating cholesterol levels in the brain [29], and it is also associated with lipid metabolism as a new causal biomarker of type 2 diabetes risk [30]. *Acsbg1* converts long-chain fatty acids into ATP and phospholipids, triglycerides, and cholesterol esters, and has been linked to obesity-driven tumor progression [31]. UDP glucuronosyltransferases (UGTs) regulate bile acid metabolism, affecting inflammatory pathology via FXR-FGF15 signaling [32]. *Pnlip*, one of the genes that codes for a key enzyme of glyceride metabolism, can enter visceral adipocytes and mediate lipotoxic systemic damage through the production of inhibited mitochondrial long-chain non-esterified fatty acids [33]. The biomarkers for the pathways of amino acid metabolism are *Aldh18a1*, *Tdo2*, *Hnmt*, *Inmt*, *Cps1*, *Ass1*, *Arg1*, *Gpt* and *Got1*. *Aldh18a1*, a key player in proline biosynthesis, drives hepatocellular carcinoma cell proliferation yet remains low in normal tissues [34]. TDO, encoded by the *Tdo2* gene, is the major hepatic enzyme regulating the catabolism of dietary tryptophan [35]. Histamine N-methyltransferase (*Hnmt*) regulates histamine metabolism, influencing low-fat food intake efficiency and potentially weight management by modulating dietary fat preferences [36]. *Inmt* is involved in tryptophan metabolism, and it has been shown that *Inmt* is strongly correlated with cognitive function, and changes in its expression are associated with obesity-related cognitive decline [37]. Arginase hydrolyzes arginine to ornithine and urea. ARG1 is the highly expressed arginase isoform in hepatocytes, and it has been shown that the expression of urea cycle enzymes is generally lower in the livers of high-fat-diet obese mice with *Cps1*, *Ass1*, and *Arg1* compared with controls fed regular chow [38]. *Gpt2* is an endoplasmic reticulum stress-activated transcription factor, and in vivo or hepatic knockdown of *Gpt2* was found to have no effect on the in vivo glucose concentrations in lean mice, but inhibiting *Gpt2* expression was found to alleviate hyperglycemia in db/db mice [39]. *Got1* is mainly involved in the reversible reaction between aspartic acid and α-ketoglutaric acid in the generation of oxaloacetic acid and glutamic acid in the cytoplasmic matrix. This process is critical for preserving cellular redox balance and supporting tumor cell proliferation, making *Got1* a potential molecular target for anticancer therapy [40]. The transcriptomic data showed that amino acid and lipid metabolism served as the principal axes of energy metabolism. The findings suggest that SCP may play a hepatoprotective role by regulating the metabolic function of the liver, and that by affecting these two pathways, the metabolic health of the host can be improved.

Using Spearman’s correlation, we found that *Faecalibaculum*, *Coriobacteriaceae_UCG-002*, and *Bifidobacterium* exhibited significant positive correlations with key genes regulating amino acid and lipid metabolism. Extensive research has demonstrated the safety profile and therapeutic potential of *Bifidobacterium* as a probiotic for human microbiota. Elevated abundance of *Bifidobacterium* species can contribute to intestinal homeostasis through a dual mechanism: preservation of gut barrier integrity and suppression of pathogenic bacterial colonization, thereby reducing metabolic endotoxin accumulation [41]. Particularly noteworthy are findings from animal studies demonstrating *Bifidobacterium*’s immunomodulatory effects in high-fat-diet models, where it can attenuate inflammatory responses and reduce endotoxin levels through the regulation and repair of T and B lymphocyte functions. Recent research indicates a strong link between *Bifidobacterium* colonization patterns and adiposity regulation, underscoring its possible influence on body fat modulation [42]. *Faecalibaculum* maintains the stability of the intestinal environment through the production of short-chain fatty acids, which help to enhance intestinal barrier function and reduce inflammation [43]. Emerging evidence also suggests that Coriobacteriaceae family members, particularly *Coriobacteriaceae_UCG-002*, serve as crucial regulators in maintaining gut microbiota homeostasis and modulating metabolic-immune interactions. Experimental studies utilizing dextran sulfate sodium (DSS)-induced colitis murine models have demonstrated a significant reduction in *Coriobacteriaceae_UCG-002* abundance, which exhibits a strong inverse correlation with pro-inflammatory cytokine levels in both intestinal and cerebral tissues [16]. These findings indicate a potential active role of increased taxa abundance in activating amino acid and lipid metabolism pathways.

Comprehensive multi-omics analyses revealed interactions between differentially expressed genes (DEGs) and differential metabolites through transcriptomic and metabolomic profiling. KEGG pathway integration revealed enrichment of two differential metabolites (citrulline and L-arginine) and five DEGs (*Cps1*, *Gpt*, *Ass1*, *Arg1*, and *Got1*) in arginine biosynthesis. The arginine biosynthesis pathway plays a key role in the regulation of body immunity. It also contributes crucially to both innate and adaptive immune responses through the synergistic action of multiple mechanisms, providing an important safeguard for the body to resist the invasion of pathogens and to maintain immune homeostasis [44]. Baicalin can ameliorate multidrug-resistant *Pseudomonas aeruginosa*-induced lung inflammation in rats through arginine biosynthesis [45]. The combined transcriptomic and metabolomic analyses confirmed that SCP exerts beneficial effects primarily by regulating arginine biosynthesis pathway, suggesting that SCP supplementation could offer tangible benefits for human health, particularly in populations with compromised immunity or metabolic dysregulation. Translating these findings into practical applications will require carefully designed human studies to establish optimal dosage forms, evaluate bioavailability, and validate the observed effects on systemic arginine metabolism and immune parameters [46]. Future clinical trials should specifically investigate SCP’s potential to maintain immune homeostasis and support metabolic health through this conserved nutrient-sensing pathway, potentially offering a dietary strategy to complement existing therapeutic approaches.

## 5. Conclusions

This study showed that the intake of dietary SCP increased beneficial bacteria and decreased harmful bacteria, while also reducing the Firmicutes-to-Bacteroidota ratio associated with obesity. This ratio was also found to be correlated with epididymal fat mass through Spearman’s correlation analysis, suggesting that SCP’s health benefits may have significant potential for preventing fat accumulation. Furthermore, gut microbiota were associated with genes and metabolites linked to amino acid and lipid metabolism. Combined metabolomic and transcriptomic analyses showed that the beneficial effect of SCP was primarily mediated through the regulation of arginine biosynthesis. These results highlight the bioactivity of SCP. Our findings provide a theoretical foundation for advancing SCP’s utilization and shed light on its potential as a health-promoting functional ingredient.

## Data Availability

The original contributions presented in this study are included in the article. Further inquiries can be directed to the corresponding authors.
